# Machine Learning‐Assisted Prediction of State of Health in Lithium Metal Batteries with Electrochemical Impedance Spectroscopy

**DOI:** 10.1002/smsc.202500277

**Published:** 2025-07-30

**Authors:** Jinsoo Yoon, Seoyoung Chae, Chaeyoung Jeong, Minju Lee, Sohui Jang, Kyoohee Woo, Hyungmin Cho, Wooseok Yang

**Affiliations:** ^1^ School of Chemical Engineering Sungkyunkwan University 2066 Seobu‐ro, Jangan‐gu Suwon 16419 Republic of Korea; ^2^ Department of Computer Science and Engineering Sungkyunkwan University 2066 Seobu‐ro, Jangan‐gu Suwon 16419 Republic of Korea; ^3^ Department of Artificial Intelligence Sungkyunkwan University 2066 Seobu‐ro, Jangan‐gu Suwon 16419 Republic of Korea; ^4^ Department of Advanced Battery Manufacturing Systems Korea Institute of Machinery & Materials (KIMM) 156 Gajeonbuk‐ro, Yuseong‐gu Daejeon 34103 Republic of Korea; ^5^ SKKU Institute of Energy Science and Technology (SIEST) Sungkyunkwan University Suwon 16419 Republic of Korea

**Keywords:** artificial intelligence, electrochemical impedance spectroscopy, Li‐metal batteries, machine learning

## Abstract

Electrochemical impedance spectroscopy (EIS) offers a nondestructive means of diagnosis for the battery's state of health (SoH). However, traditional equivalent circuit‐based approaches—relying on extensive modeling and fitting of complex EIS data such as real and imaginary impedance components, phase shift, and frequency—are time‐consuming and heavily dependent on expert interpretation, which can compromise reliability. In this context, artificial intelligence‐based models present a faster and more reliable alternative for interpreting EIS data. These models can uncover hidden patterns and parameters that may be overlooked by human experts, thereby enabling more accurate prediction of the battery's SoH. In this study, four machine learning algorithms are employed to predict the SoH of lithium metal batteries based on EIS data, achieving predictive accuracies exceeding 95%. Feature importance analysis indicated that phase shift—an often underutilized parameter in conventional EIS interpretation—plays a critical role in the SoH prediction process. Furthermore, the analysis enabled the attribution of specific EIS features to their corresponding electrochemical phenomena, thereby elucidating the physical basis of the model predictions. The resulting models exhibit high precision in forecasting battery discharge capacity and diagnosing degradation mechanisms, demonstrating their potential as powerful tools for advancing battery diagnostics and performance optimization.

## Graphical abstract

This study investigates the artificial intelligence‐driven method for predicting the state of health of lithium metal batteries based on electrochemical impedance spectroscopy (EIS) data. Four machine learning algorithms achieve accuracy exceeding 95%, identifying phase shift as a critical feature for prediction and establishing a connection between EIS‐derived parameters and underlying electrochemical degradation mechanisms.

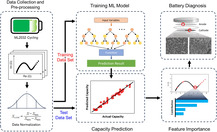

## 1 Introduction

With the global increase in energy demand, extensive research efforts have focused on improving the capacity of lithium‐ion batteries through advancements in the electrode structure and materials.^[^ ^1^, ^2^, ^3^, ^4^ ^]^ Among the proposed strategies, the implementation of alternative anodes has attracted significant attention, with lithium metal batteries (LMBs) considered promising candidates. Conventional lithium‐ion batteries utilize graphite as the anode material and rely on a reversible intercalation mechanism whereby lithium ions are inserted between the graphene layers during charging. In contrast, LMBs employ metallic lithium as the anode, on which lithium ions are directly deposited as metallic lithium. This fundamental difference endows lithium metal with a theoretical specific capacity of 3850 mAh g^−1^, which is approximately an order of magnitude greater than that of graphite (372 mAh g^−1^). This exceptional high energy density makes lithium metal a promising anode material for the development of next‐generation high‐capacity energy storage systems. However, LMBs are more susceptible than conventional lithium‐ion batteries to the formation of lithium dendrites because of the elevated electric field at the electrode surface during lithium deposition, which poses serious safety and performance challenges.^[^ ^5^, ^6^ ^]^ The formation of lithium dendrites can lead to the breakdown of the battery separator and cause short circuits.^[^ ^7^ ^]^ These safety challenges represent major barriers to the large‐scale commercialization and practical deployment of LMBs. Given the inherent risks associated with lithium metal anodes, particularly those related to dendrite formation and thermal instability, accurate prediction of the state of health (SoH) and reliable identification of degradation mechanisms are essential to ensure operational safety and extend the battery lifespan.

Electrochemical impedance spectroscopy (EIS) has gained attention as a nondestructive diagnostic tool for batteries.^[^ ^8^ ^]^ EIS is an analytical technique in which a sinusoidal voltage or current is applied to an electrochemical system over a range of frequencies and the resulting response is measured. This method enables the deconvolution of complex electrochemical processes by characterizing the frequency‐dependent impedance, thereby providing insights into the reaction kinetics, charge‐transfer resistance, and interfacial capacitance within the system.^[^ ^9^ ^]^ When EIS is applied to batteries, the impedance spectrum reveals the resistances arising from internal interfaces, charge storage, and charge transfer within the battery.^[^ ^10^ ^]^ Therefore, by analyzing the impedance spectrum as a function of battery cycling, the key factors contributing to battery degradation can be identified. Equivalent circuit (EC) models have been widely used to interpret EIS data.^[^ ^11^ ^]^ This approach represents the internal electrochemical processes of a battery as a combination of resistive and capacitive elements within an electrical circuit, enabling the association of specific components with distinct interfacial phenomena and providing a framework for electrochemical interpretation. However, the accuracy of EC‐based analyses is often limited by the overlapping of signals arising from electrochemical processes with similar relaxation time constants, which complicates the deconvolution of individual contributions. Additionally, EIS measurements yield a wide array of parameters, including the impedance magnitude, real (resistance) and imaginary (reactance) components, phase shift, and frequency, which further increases the complexity of the analysis. Consequently, the EC modeling approach is both time‐consuming and heavily reliant on expert interpretation, making consistent and accurate SoH prediction challenging.

Given the limitations of EC methods, data science approaches capable of identifying patterns within complex high‐dimensional datasets offer a promising new framework for impedance analysis and SoH prediction. In particular, artificial intelligence (AI), a key tool in data science, has been widely applied to various aspects of battery analysis, including state of charge and lifetime prediction.^[^ ^12^, ^13^, ^14^ ^]^ Despite the great potential of impedance data, their use has largely been limited to serving as training data for lifetime prediction models, rather than being leveraged for direct battery state diagnostics.^[^ ^15^ ^]^ Furthermore, existing approaches have not adequately addressed the interpretability of the data.^[^ ^16^ ^]^ This is primarily due to the challenges associated with integrating domain knowledge from both data science and EIS, which is essential for building accurate and interpretable SoH prediction models. This challenge is even more pronounced in the case of LMBs, where studies focusing on impedance spectrum analysis are notably scarce.

In this study, machine learning was applied to the impedance spectrum of LMBs to enable SoH prediction and facilitate the interpretation of the underlying physical mechanisms using feature importance analysis. Impedance spectra were obtained from lithium metal alloy coin cells during charge–discharge cycling and were used to train the machine learning models. Models capable of predicting the battery SoH with an accuracy exceeding 95% based solely on impedance spectrum data were identified. The feature importance extracted from the high‐performing models revealed the specific parameters that the models considered the most influential in determining the SoH. These parameters were then used to interpret the physical origins within the impedance spectrum that significantly affect the SoH, demonstrating that the phase shift, often overlooked in conventional EC methods, can serve as a key indicator in LMB SoH prediction. Our work establishes a new impedance spectrum interpretation framework by identifying critical parameters that contribute to accurate SoH prediction, offering insights that are difficult to obtain using EC methods.

## 2 Results and Discussion

### 2.1 Challenges in SoH Prediction Using Impedance Spectrum

The LMB used for impedance data collection was a Maxell ML2032, which features a lithium–aluminum alloy anode. Lithium–aluminum alloy batteries are LMBs designed to address the instability of pure lithium metal anodes and improve their cyclability.^[^ ^2^ ^]^ Although the theoretical capacity of the lithium–aluminum alloy (993 mAh g^−1^) is lower than that of pure lithium metal, its relatively uniform lithium deposition enhances its stability, making it suitable for application in commercial cells.^[^ ^17^ ^]^

**Figure 1:**
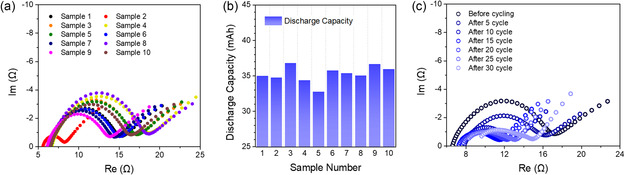
Differences in the a) impedance spectra and b) discharge capacities of 10 random fresh ML2032 coin cell samples before cycling. c) Change in the impedance spectrum of the ML2032 coin cell with cycling.

**Figure** **1**a presents the impedance spectra of 10 randomly selected coin cells in their precycling states from 105 LMB coin cell samples. Each impedance spectrum was measured over a frequency range from 100 kHz to 50 mHz, with each spectrum consisting of data from 54 distinct frequency points. When analyzing the impedance spectrum using the EC method, the semicircular features observed in the Nyquist plot are typically attributed to individual parallel resistor–capacitor (RC) elements. These elements represent distinct electrochemical processes arising primarily from interfacial phenomena. The specific configuration of the RC elements in the EC model was determined based on the physical interpretation of the internal impedance components of the battery. In general, the number of RC elements employed corresponds to the number of clearly distinguishable semicircles in the impedance spectrum (Figure S1, Supporting Information). The diameter of the semicircle corresponds to the magnitude of the interfacial resistance, with larger semicircles indicating higher resistance. Notably, the Nyquist plots reveal considerable variation in the semicircle size across the cells, despite all samples being of the same battery type. This variability highlights a key limitation of the EC‐based interpretation: its sensitivity to cell‐to‐cell variation and the potential for misleading conclusions, particularly when assessing battery conditions solely based on semicircle dimensions. The comparison of the impedance spectrum characteristics with the discharge capacity of the 10 coin cells shown in Figure 1b highlights the challenges associated with SoH prediction using the EC method. The EC method, which estimates the internal resistance based on the diameter of the semicircle in the Nyquist plot, implies that cells exhibiting larger semicircles and thus higher interfacial resistance should exhibit lower discharge capacities. However, the experimental results reveal no consistent or direct correlation between the semicircle size and the actual discharge capacity of the cell. This discrepancy suggests that reliance on semicircle‐based resistance estimation alone may be insufficient to evaluate the battery performance or predict the SoH accurately. The necessity of data science‐based analysis also appears in the impedance spectrum characteristics of LMBs. In batteries employing graphite anodes, EC‐based analysis may offer a relatively straightforward approach to predict the SoH, as both the decline in discharge capacity and the rise in internal resistance generally follow linear trends.^[^ ^18^ ^]^ In contrast, for LMBs, the correlation between the changes in the semicircle size and the number of cycles is less clear, as shown in Figure 1c. This ambiguity complicates the establishment of a direct link between the increase in specific interface resistance and the decrease in discharge capacity, underscoring the need for alternative analytical approaches.

### 2.2 Machine Learning‐Assisted Prediction of Discharge Capacity

During the impedance spectrum extraction phase, a total of 695 tabular impedance data, excluding abnormal and noninterpretable data, were collected before cycling and every five cycles from 105 ML2032 coin cells. As explained earlier, the data for eight categories were extracted for each of the 54 frequencies per coin cell. The data value distribution of each data category for the entire tabular impedance dataset is shown in Figure S2, Supporting Information.

**Figure 2:**
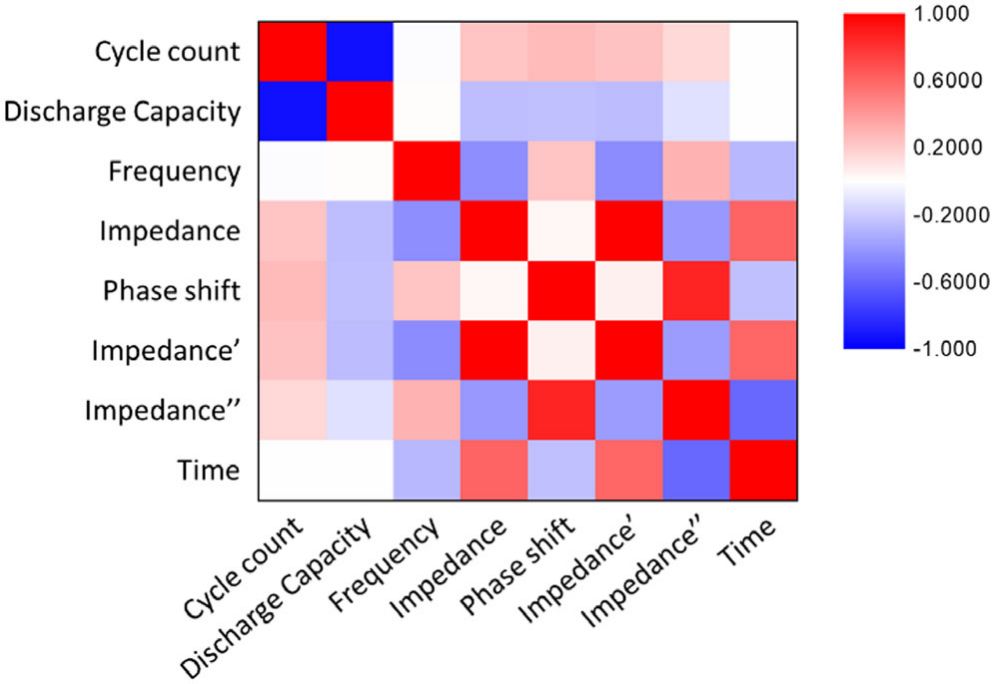
Correlation analysis of impedance spectrum data.

Using the dataset, a correlation analysis was conducted to validate the existence of a relationship between the discharge capacity and impedance spectrum, as briefly discussed earlier when describing the Nyquist plot, and to explore the correlations among the other data categories. The heatmap in **Figure** **2** illustrates the correlation analysis results between the discharge capacity and each column of the impedance dataset. The figure illustrates the Pearson correlation coefficient, a statistical measure that quantifies both the strength and direction of the linear relationship between multiple variables, presented in matrix form. This measure of common variation is normalized by dividing the covariance between variables by the standard deviation of each variable. In the heatmap, if the color of the cell where the category rows and columns intersect is red, the correlation between the two categories is positive, whereas if it is blue, the correlation is negative. In addition, the stronger the correlation, the higher the color saturation. The specific values of the correlation coefficients, which show the correlation strength between the data categories, are presented in Figure S3, Supporting Information. The goal of this study was to extract data from the impedance spectrum that significantly contributes to the decision‐making of the AI model and to analyze the electrochemical causes of these data. Therefore, the decision‐making process of the AI model should focus on the relationships between the impedance data categories, which can be represented in the Nyquist plot (impedance, phase shift, impedance', and impedance”), and the discharge capacity. The analysis results reveal a weak correlation between these variables, which cannot intuitively be observed in the Nyquist plots. However, the cycle count exhibits a strong negative correlation with the discharge capacity. The strong correlation is because, regardless of the type or extent of the electrochemical causes, the battery capacity decreases as the charging and discharging cycles continue. However, the correlation strength of the cycle count is much higher than those of the four mentioned data categories. In this case, when the cycle count data category is included in the AI training dataset, the SoH prediction relies on the cycle count rather than the data from the impedance spectrum. To prevent the dependence on cycle count, the cycle count column was excluded from the tabular impedance data.

After selecting the data columns based on the correlation analysis, an AI‐based model suitable for predicting the SoH using tabular impedance data was chosen. Before training, the values within each data category were normalized for preprocessing: $x_{scaled}=\frac{x-x_{min}}{x_{max}-x_{min}}$, where $x_{min}$ is the minimum value and $x_{max}$ is the maximum value of each data category. The impedance data were divided into training and test sets of 556 and 139 impedance data, respectively. In this experiment, tabular data were used to maximize the predictive utility of the parameters contained within each data point. Algorithms such as the categorical boosting (CatBoost), light gradient boosting machine (LightGBM), random forest, and extreme gradient boosting (XGBoost) algorithms have demonstrated superior performance when applied to tabular data.^[^ ^19^ ^]^ Based on these findings, the top four algorithms were applied to SoH prediction. Detailed explanations of the four algorithms are provided in Table S1, Supporting Information. Although deep learning models based on artificial neural networks have been introduced in the literature, a machine learning approach was more suitable for the objectives of our study. Unlike image or text data, which have clear spatial relationships, tabular data are influenced by the interactions among multiple variables. In such cases, deep learning models struggle to interpret data structures effectively, making them less suitable for tabular datasets.^[^ ^20^ ^]^ In addition, the complexity of the weight trees generated during the prediction process in deep learning models, such as long short‐term memory model, is significantly higher than that in machine learning models, known as the black box problem, causing difficulty in determining the contributions of individual data points.^[^ ^21^, ^22^ ^]^ This characteristic is inconsistent with the objective of this study, which was to analyze the causes of battery degradation by interpreting the criteria for the prediction of AI‐based models using feature importance. Therefore, four machine learning models (CatBoost, XGBoost, random forest, and LightGBM) that demonstrate high accuracy and enable the extraction of the feature importance for more interpretable analysis were employed.

**Figure 3:**
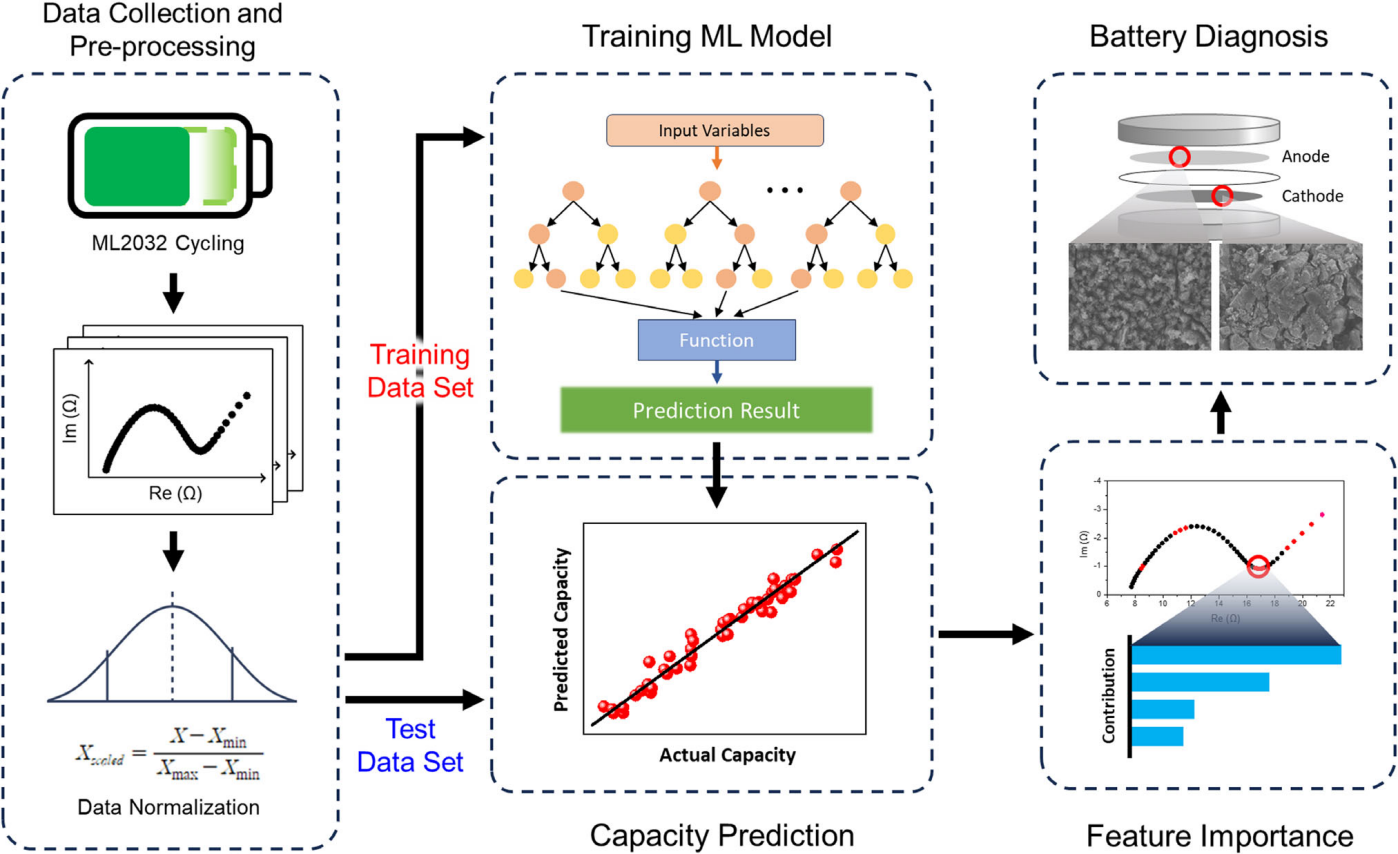
Flow diagram of the whole process for SoH prediction and interpretation of the degradation mechanism using a machine learning model.

**Figure** **3** provides a flow diagram of the entire process of predicting the discharge capacity and extracting the causes of degradation through feature importance analysis with selected machine learning models from the impedance spectra of lithium–aluminum alloy batteries. Our original datasets, code, and raw results are available at https://github.com/pcc1016/Prediction‐of‐Discharge‐Capacity‐in‐Lithium‐Metal‐Batteries.git. Before the feature importance analysis, capacity prediction was performed to evaluate the reliability of the selected machine learning models. A summarized explanation is provided, and the parameters of the four machine learning models used in the capacity prediction experiment are described below.

#### 2.2.1 XGBoost

XGBoost is an advanced implementation of the gradient‐boosting framework that improves each subsequent model based on the errors of the previous model.^[^ ^23^ ^]^ For the training parameters of the XGBoost model in this study, the learning rate was set to 0.1 and the number of trees used for training was 100.

#### 2.2.2 CatBoost

CatBoost is a gradient boosting‐based machine learning model with the “ordered boosting” method, where the model calculates the loss for each data point without using that specific data point in training to avoid overfitting to the training set.^[^ ^24^ ^]^ For the training parameters of the CatBoost model in this study, the learning rate was set to 0.1 and the number of trees used for training was 1000.

#### 2.2.3 Random Forest

The random forest model is an ensemble learning algorithm, meaning that it trains on a subset of data and aggregates the predictions to make a final prediction.^[^ ^25^ ^]^ In this study, 100 trees were used for training.

#### 2.2.4 LightGBM

LightGBM is a gradient‐boosting framework with gradient‐based one‐sided sampling and an exclusive feature‐bundling method that improves efficiency by reducing the number of data instances and features.^[^ ^26^ ^]^ For the training parameters of the Light GBM model in this study, the learning rate was set to 0.05, and the number of trees used for training was 100.

For accurate SoH prediction and meaningful impedance analysis, the scope of the single dataset to be used to train the selected machine learning model must be determined. The tabular impedance data obtained through data acquisition consisted of seven data category columns, excluding the cycle count, and 54 rows representing the measured frequencies. Because of the nature of EIS, the frequency data category is associated with the impedance of the electrochemical reactions that occur at specific reaction rates at each frequency. Therefore, even when a single data entry is defined as one row within the tabular impedance data, it can still provide interpretable insights into the individual resistance elements. However, when training was performed using a single row as the data input, the prediction accuracy dropped below 90% (Figure S4, Table S2, Supporting Information). Another approach is to consider single‐tabular impedance data as “single data.” Given that one tabular impedance data contain sufficient information to generate one Nyquist plot, the number of data points contributing to the SoH prediction increases, thereby improving the accuracy of the SoH prediction. This approach also offers advantages from the perspective of impedance analysis. The impedance at a specific frequency in the impedance spectrum is not caused by a single factor, rather, it results from the responses of various electrochemical reactions. By considering the connections between the impedances at the previous and subsequent frequencies, a deeper analysis of the underlying causes is possible. Most importantly, this approach aligns with the experimental goal of using AI to derive new criteria from large amounts of information, which could not be achieved through earlier analyses.

**Figure 4:**
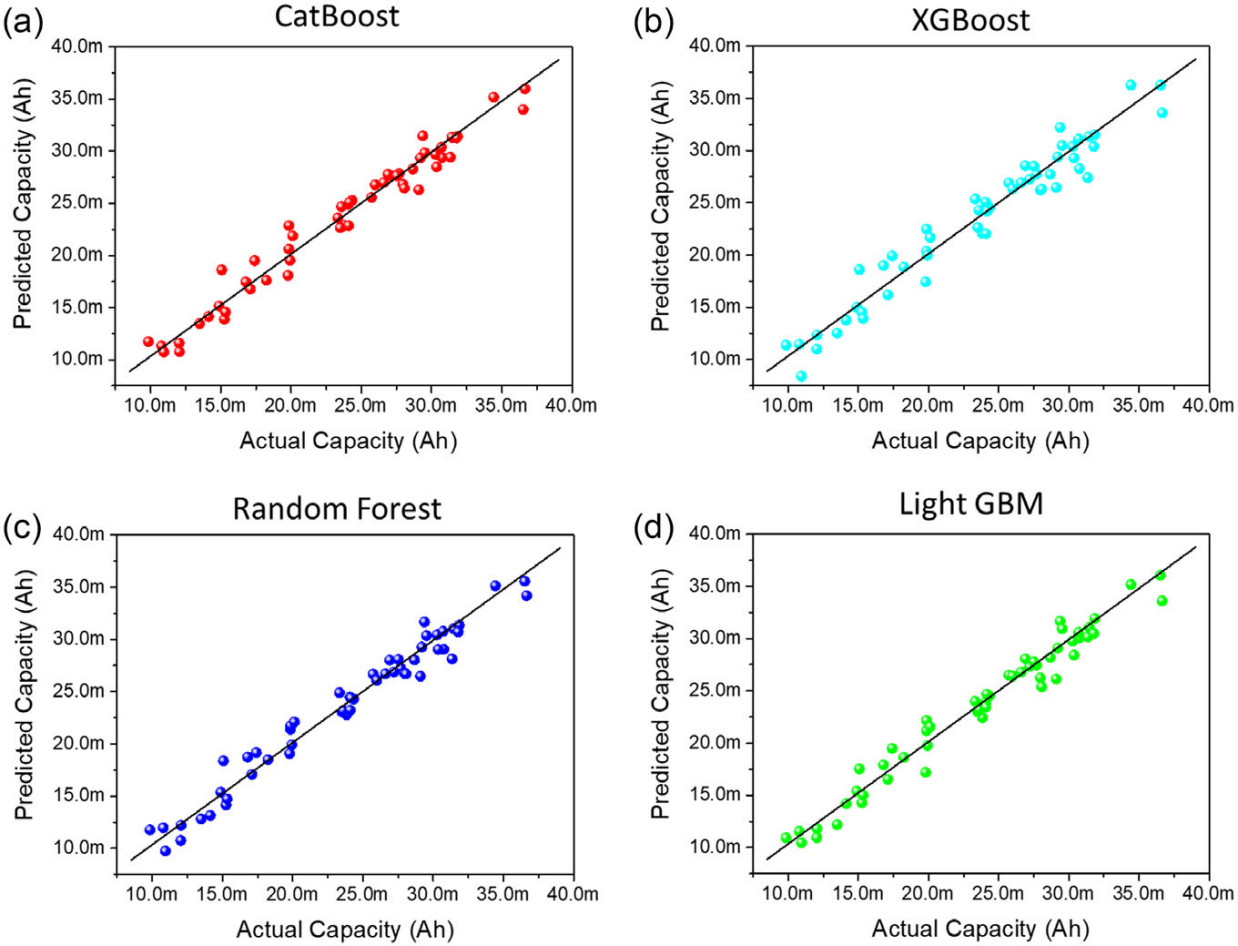
Graph of actual capacity versus predicted capacity of ML2032 obtained using a) CatBoost, b) XGBoost, c) random forest, and d) LightGBM, trained using single data as tabular impedance data.

The capacity prediction performance when the tabular impedance data were used as single data and trained with the four selected machine learning models is shown in **Figure** **4**. This figure confirms that all machine learning models can predict the capacity with minimal errors from the actual discharge capacity, solely based on the impedance spectrum data points, without the involvement of the cycle count data category. **Table** **1** provides the error rates in the capacity prediction, demonstrating that the machine learning models used in this experiment can achieve over 95% accuracy, confirming their practical applicability.

**Table 1:** Prediction accuracy of machine learning models.

Machine learning model	Mean‐squared error	R‐squared
XGBoost	2.2710 × 10^−6^ (±0.997 × 10^−6^)	0.9471 (±0.0187)
CatBoost	2.5815 × 10^−6^ (±0.8636 × 10^−6^)	0.9395 (±0.0169)
Random forest	1.9944 × 10^−6^ (±0.6188 × 10^−6^)	0.9533 (±0.0105)
LightGBM	2.2535 × 10^−6^ (±1.035 × 10^−6^)	0.9476 (±0.0195)

### 2.3 Feature Importance Analysis

The discharge capacity prediction results demonstrated a high prediction accuracy of over 93% across the four machine learning models. To identify the data points within the impedance spectrum data that were the most heavily weighted in predicting the discharge capacity of each of the four machine learning models, feature importance analysis was conducted.

#### 2.3.1 XGBoost

For the XGBoost model in this experiment, the gain was used to calculate the data contribution of each feature of the model. The gain measures the average improvement in accuracy brought about by a feature in the branches it splits. This factor represents an improvement in the objective function when splitting the data based on that feature.

#### 2.3.2 CatBoost

The following function was used to calculate the feature importance of CatBoost model

(1) $$Feature\;importance\;=\;\sum_{tree}\left(c_{1}{\left(v_{1}-avr\right)}^{2}-c_{2}{\left(v_{2}-avr\right)}^{2}\right)$$

where $c_{1}$ and $c_{2}$ are the weights, avr is the average weight value, and $v_{1}$ and $v_{2}$ are the leaf values.

#### 2.3.3 Random Forest

To examine the data contribution of each feature, the impurity‐based feature importance was calculated by using Gini impurity and applying the following equation

(2) $$Feature\;importance\;=\;1-\sum_{i=1}^{n}{\left(p_{i}\right)}^{2}$$

where $p_{i}$ is the probability of class *i*.

#### 2.3.4 LightGBM

To determine feature importance in LightGBM model, the “split” method was employed. This approach calculates the feature importance by counting the frequency at which each feature is used for node splitting when the model achieves its peak performance.

**Figure 5:**
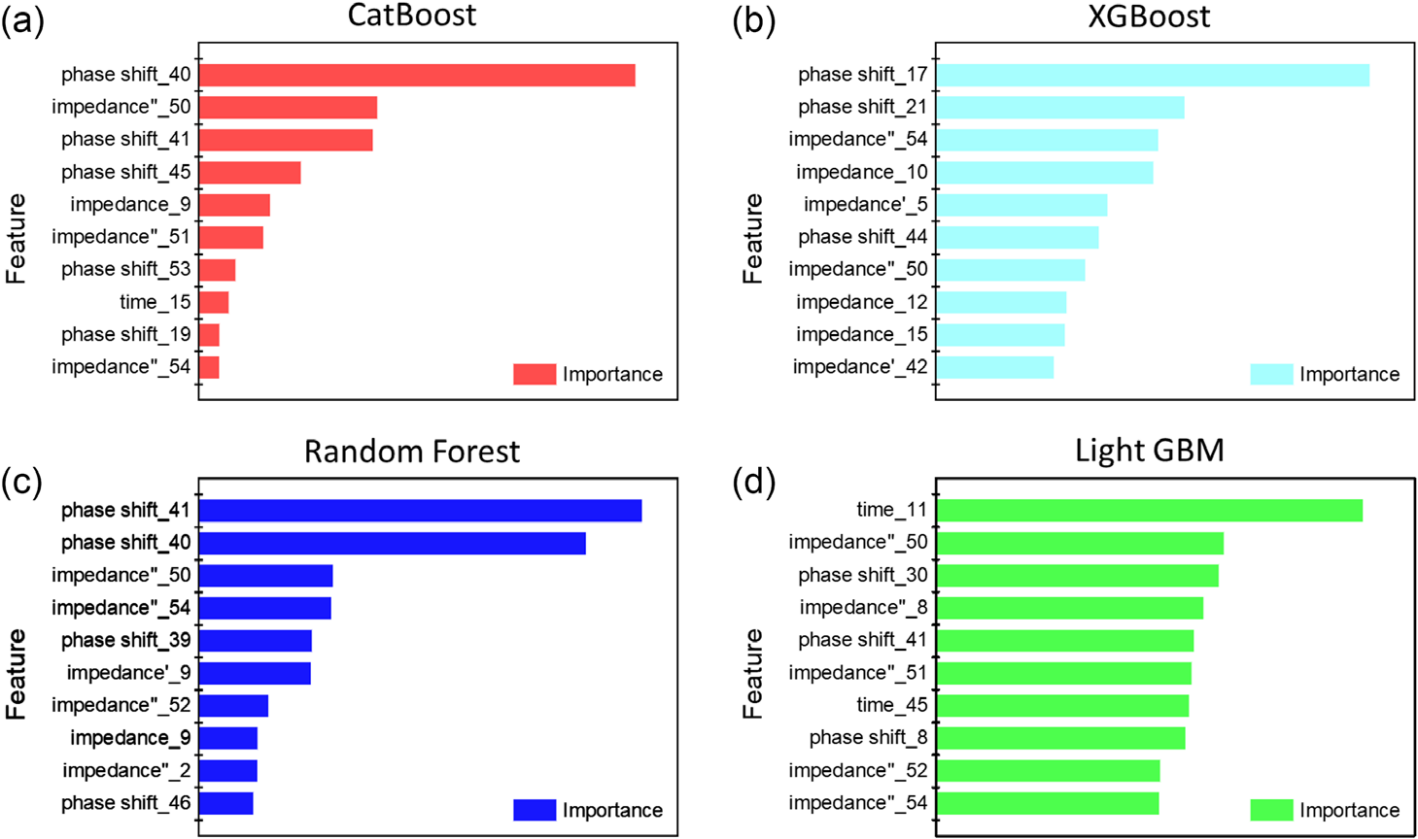
Data contribution for predicting the specific capacity of ML2032 in the a) CatBoost, b) XGBoost, c) random forest, and d) LightGBM models.

**Figure** **5** illustrates the feature importance analysis results derived from each machine learning model. The numbers associated with each feature correspond to the row indices in the tabulated impedance data, where the lower numbers represent measurements at higher frequencies. The frequencies of the applied AC voltages corresponding to each measured data point are listed in Table S3, Supporting Information. As discussed previously, the conventional EC method interprets the interfacial resistance based on the diameter of the semicircle in the Nyquist plot. Therefore, the EC method assesses the SoH of batteries based primarily on the x‐value (the real part of the impedance) corresponding to the diameter of the impedance semicircle. However, in LMBs, irregular changes in the x‐value hamper accurate SoH estimation. In contrast, AI‐based analysis of EIS data offers a more comprehensive and nuanced interpretation. Feature importance algorithms identify the phase shift at certain frequencies as a key parameter in predicting the discharge capacity, a factor that is not directly emphasized or easily visualized in traditional EC analysis. Notably, the phase shift values of the 40th and 41st data points show high importance across multiple models, including random forest, CatBoost, and LightGBM. In addition, LightGBM highlights a contribution from the 30th point, and CatBoost highlights the 45th point. XGBoost emphasizes the phase‐shift values of the 17th and 21st data points. The phase shift, defined as the angle between the x‐axis and the line connecting the origin to a given point on the Nyquist plot, is typically used only indirectly in EC modeling owing to its contribution to the imaginary part of the impedance. However, it does not serve as a standalone criterion for interpreting impedance behavior. Machine learning models, unconstrained by traditional circuit analogies, recognize the phase shift as a significant predictor of electrochemical behavior. This finding highlights a major advantage of AI‐driven approaches: the ability to detect and leverage subtle complex patterns in high‐dimensional EIS data that are often overlooked in conventional analyses. These results suggest that the phase shift may serve as a previously underappreciated but powerful indicator of the degradation processes in LMBs.

**Figure 6:**
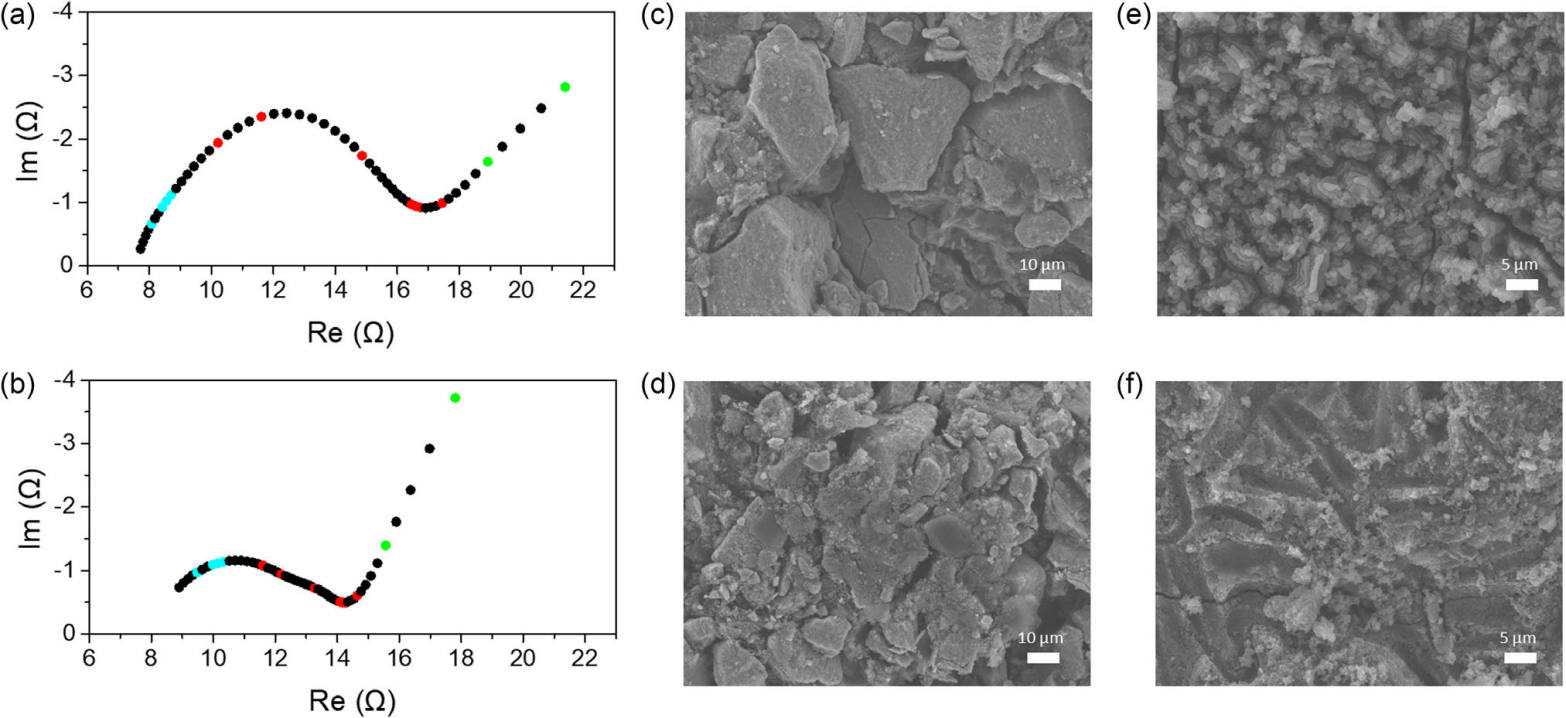
Impedance spectra of a) a fresh cell and b) an aged cell. The colored data points are the top five contributing data points from each machine learning model. SEM images of the MnO_2_ cathodes of c) a fresh cell and d) an aged cell that was cycled for 30 cycles. SEM images of the lithium–aluminum anodes of e) a fresh cell and f) an aged cell.

To elucidate the relationship between feature importance in the machine learning models and the underlying electrochemical processes, the top five contributing data points identified by each model (13 data points in total after removing duplicates) were highlighted in the Nyquist plots (**Figure** **6**a,b). The positions of the top five contributing data points in each machine learning model can be identified in the impedance spectrum, shown in Figure S5, Supporting Information. The data points are color‐coded based on their associated frequency domains: high frequency (cyan points), mid‐frequency (red points), and low frequency (green points). The EIS signals within each frequency domain correspond to distinct electrochemical processes, each characterized by different relaxation times within the battery.^[^ ^10^ ^]^ Scholars widely recognize that the mid‐frequency domain poses significant challenges for SoH prediction of LMBs using the EC method. The impedance in the mid‐frequency domain primarily originates from the solid–electrolyte interphase (SEI) on the anode and charge transfer at the interface between the active material and the electrolyte. As previously discussed, interpreting the semicircle observed in the mid‐frequency range using the EC method does not reveal a clear correlation between the interfacial resistance and discharge capacity. However, regardless of the type of model, all four machine learning models offer complementary interpretive perspectives by highlighting the phase shift as a critical indicator in this frequency domain (red points). For example, the XGBoost model identifies the phase‐shift values near the local maximum of the semicircle (especially phase shift_17 and phase shift_21) as highly important features. Meanwhile, the CatBoost, random forest, and LightGBM models assign high feature importance to the phase‐shift values located near the local minimum between the mid‐ and low‐frequency domains (especially phase shift_40 and phase shift_41).

**Figure 7:**
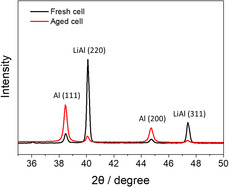
Comparison of XRD patterns for the lithium–aluminum anode between fresh and aged cells.

One possible explanation for this phenomenon is the distinct degradation mechanism of LMBs, which involves MnO_2_ cathode fragmentation and the formation of lithium plating and dead lithium. As shown in Figure 6c,d, the active material particles in the MnO_2_ electrode of the aged cell are significantly smaller than those in the fresh cell, indicating that particle fragmentation occurs due to volume changes during cycling. The fragmentation of the cathode active material increases the total surface area available for lithium‐ion intercalation. The expansion of the electrochemically active surface promotes parallel pathways for ion transfer, effectively reducing the size of the semicircle associated with the charge‐transfer impedance in the Nyquist plot.^[^ ^27^ ^]^ In lithium–aluminum alloy anodes, the degradation mechanisms differ from those of the cathode. During cycling, lithium plating and lithium–aluminum alloy formation dominate the ion storage processes at the anode–electrolyte interface. These mechanisms increase the likelihood of dendritic growth and formation of dead lithium.^[^ ^28^ ^]^ The scanning electron microscope (SEM) images in Figure 6e,f reveal that whereas the fresh cells display a well‐defined skeleton structure of lithium–aluminum alloys, the aged cells exhibit layered structures owing to lithium plating and dead lithium accumulation. Additionally, the X‐ray diffraction (XRD) patterns of the postcycling electrodes show a significant decrease in the lithium–aluminum peak and an increase in the unalloyed aluminum peak in the aged cells, indicating lithium consumption and localized concentration at the interface (**Figure** **7**). Excess lithium on the surface alters the composition of the SEI layer, increasing the metallic content, and consequently modifying the charge‐transport mechanism. This shift leads to the appearance of an SEI interfacial impedance at frequencies higher than those typically expected.^[^ ^11^, ^29^ ^]^ Consequently, the semicircle associated with the SEI impedance shifts toward the high‐frequency domain, whereas the semicircle corresponding to the charge‐transfer impedance, which previously overlapped with the SEI impedance, diminishes in size. Therefore, the Nyquist plot transitions from a single merged semicircle to two distinct deconvoluted semicircles. The machine learning models captured this complex behavior by assigning high importance to the data points around the local maximum and minimum in the Nyquist plot, where the distortion of the semicircle reflects a reduction in the phase shift. This result indicates that the deformation of the semicircle, which is a subtle feature often overlooked by the EC method, can be effectively identified using machine learning. The results demonstrate the ability of the model to uncover electrochemical degradation patterns through phase‐shift analysis, an aspect that is not readily accessible via conventional EC‐based approaches.

Although the machine learning models assigned high importance to the phase shift as a novel interpretive parameter in the mid‐frequency domain, data points consistent with EC method‐based interpretations were also identified in the high‐frequency (cyan points) and low‐frequency domains (green points). The high‐frequency domain is primarily associated with the inherent ohmic resistance of the electrolyte and the impedance at the current collector–electrode interface, where electron transfer occurs. In aged cells, the ohmic resistance increases compared to that of fresh cells during charge–discharge cycling.^[^ ^30^ ^]^ Additionally, the repeated volume changes of the electrodes during cycling weaken the adhesion between the current collector and electrode, leading to increased interfacial impedance.^[^ ^31^ ^]^ This phenomenon increases the impedance, impedance′, and impedance″ categories of the data, and certain data points—such as impedance_10, impedance_9, and impedance″_8—influence the predictions made by the machine learning models. The low‐frequency domain is related to lithium‐ion diffusion within the active material of the cathode. In the low‐frequency domain, the impedance appears as a diagonal line in the Nyquist plot, which is commonly interpreted as the Warburg impedance in EC modeling. This behavior corresponds to semi‐infinite diffusion of lithium ions through the active material. In the aged cells, the slope of the Warburg line with respect to the x‐axis is noticeably steeper than that in the fresh cells. This change is attributed to the fragmentation of the cathode active material, as previously discussed. As the active material becomes more fragmented, the diffusion path for lithium ions shortens, transitioning the impedance behavior from semi‐infinite (Warburg) to finite space, resembling pure capacitive behavior.^[^ ^32^ ^]^ In the Nyquist plot, the pure capacitance–impedance is represented by a nearly vertical line at low frequencies. Thus, the increased slope observed in the low‐frequency domain reflects a transition toward pure capacitance. This interpretation is also supported by the feature importance analysis from the machine learning models, where, excluding LightGBM, all three models assigned high importance to the imaginary component of the last data point in the Nyquist plot (impedance″_50 and impedance″_54). The increase in the y‐value of the last point of the impedance data, along with a steeper angle, contributes to the SoH prediction.

Thus, analyzing the importance of features for SoH prediction based on the impedance spectra of LMBs could lead to the establishment of a new framework for the electrochemical interpretation of degradation. This framework may also quantify the degradation levels and serve as a benchmark for assessing and improving the performance of new batteries.

## 3 Conclusion

This study established a machine learning‐based model to predict and analyze LMB degradation using EIS. Impedance spectrum data were extracted from commercial lithium–aluminum alloy batteries, and cycle information was excluded during AI training to develop a prediction model based solely on spectral features rather than simple charge–discharge cycles. Among the machine learning algorithms applied, CatBoost, XGBoost, random forest, and LightGBM demonstrated high accuracy (exceeding 93%) in predicting battery performance using tabular EIS data. Feature importance analysis revealed that, unlike in traditional EC interpretations, phase shifts significantly influence the degradation criteria in LMBs. The electrochemical origin of each criterion was elucidated by comparing the top five contributing features of each model. For data points where impedance and related features exhibited high importance, degradation was attributed to increases in resistance, in agreement with the EC models. Data points where the phase shift was a critical feature explained the electrode degradation through distortions and separations in the semicircles, offering a novel perspective that is undetectable by conventional models. This study confirms the ability of machine learning models to predict LMB discharge capacity with high precision and identify the causes of degradation through feature importance. Furthermore, this study presents the potential for a new approach to utilizing impedance data for performance prediction across various batteries, beyond LMBs. The results of this research, where a machine learning model introduces phase shift as a novel criterion for SoH prediction, suggest that data science‐based approaches can offer new applications for impedance data in various batteries, which were not utilized in traditional EC‐based analyses. The applicability of the model to newly developed batteries highlights its potential for advanced battery‐degradation analyses and solutions.

## 4 Experimental Section

4.1

4.1.1

##### Battery Cycling

For the experiments aimed at assessing the primary interests of capacity degradation prediction, namely, safety and practicality, an ML2032 (Maxell) with a lithium–aluminum alloy anode and MnO_2_ cathode was subjected to harsh charging and discharging conditions. To quickly observe impedance spectrum changes similar to the capacity variations caused by degradation in the battery, a total of 105 coin cell samples were charged and discharged by a WBCS3000L32 (Wonatech) in a 25 °C controlled chamber with a current density of 6.5 mA, which is 10 times higher than the recommended charge–discharge current density for the ML2032. The upper and lower cutoff voltages were set to 3.25 V and 1.5 V, respectively. During each charge–discharge cycle, the discharge and charge capacities were recorded. Although the same ML2032 coin cells were used, the initial capacities varied, and the distribution of these capacities can be observed in Figure S6, Supporting Information. Charging and discharging were repeated until the discharge capacity had decreased to 50% of the initial value.

##### EIS Measurement

Impedance measurements were performed once prior to the first cycle and subsequently every five cycles. EIS was conducted using Zennium Pro and Thales software (Zahner‐Elektrik). The potentiostat‐mode EIS was measured at the open‐circuit voltage of the coin cells across 54 frequencies ranging from 100 kHz to 50 mHz, with an AC voltage amplitude of 10 mV. Each impedance spectrum consisted of 54 data points, and each data point included the following features: cycle count, discharge capacity, frequency, impedance, phase shift, real part of the impedance (impedance′), imaginary part of the impedance (impedance″), and time of the impedance spectrum measurement (time). The data are organized in tabular form. Among the obtained data, data with abnormal impedance spectrum shape were removed.

##### Battery Disassembly and Measurement

The ML2032 was disassembled using a coin cell disassembler (Wellcos) in an argon‐filled glove box for surface analysis. An SEM (JSM‐IT800, JEOL) was used to characterize the anode and cathode surfaces of disassembled ML2032. The electrodes were prepared by coating their surfaces with platinum. XRD was performed using a D8 Discover diffractometer (Bruker).

## Conflict of Interest

The authors declare no conflict of interest.

## Declaration of generative AI and AI‐assisted technologies in the writing process

During the preparation of this work, the authors used ChatGPT to correct typographical and grammatical errors. After using this tool, the authors reviewed and edited the content as needed and take full responsibility for the content of the publication.

## Supporting information

Supplementary Material

## Data Availability

The data that support the findings of this study are available from the corresponding author upon reasonable request.
