# Supplementary material for: Machine Learning‐Assisted Prediction of State of Health in Lithium Metal Batteries with Electrochemical Impedance Spectroscopy
Source: Small Sci. 2025 Jul 30;5(11):2500277. doi: 10.1002/smsc.202500277 (PMC12622547; doi:10.1002/smsc.202500277)
Supplement: Supplementary file 1 — Supplementary Material [file SMSC-5-2500277-s001.pdf]

Supporting Information

Machine Learning-Assisted Prediction of State of Health in Lithium Metal Batteries with Electrochemical Impedance Spectroscopy

‡ *Jinsoo Yoon*[a], ‡ *Seoyoung Chae*[b], ‡ *Chaeyoung Jeong*[c], *Minju Lee*[a], *Sohui Jang*[a,d], *Kyoohee Woo*[d], *Hyungmin Cho*[b] *and Wooseok Yang*[a,e]*

a. Sungkyunkwan University, School of Chemical Engineering, Engineering Building Ⅱ, 2066 Seobu-ro, Jangan-gu, Suwon 16419, Republic of Korea
b. Sungkyunkwan University, Department of Computer Science and Engineering, 2066 Seobu-ro, Jangan-gu, Suwon 16419, Republic of Korea
c. Sungkyunkwan University, Department of Artificial Intelligence, 2066 Seobu-ro, Jangan-gu, Suwon 16419, Republic of Korea
d. Korea Institute of Machinery & Materials, 156 Gajeonbuk-ro, Yuseong-gu, Daejeon 34103, Republic of Korea
e. SKKU Institute of Energy Science and Technology (SIEST), Sungkyunkwan University, Suwon 16419, Republic of Korea

‡These authors contributed equally

*Corresponding authors
Prof. Wooseok Yang (wooseok.yang@skku.edu)

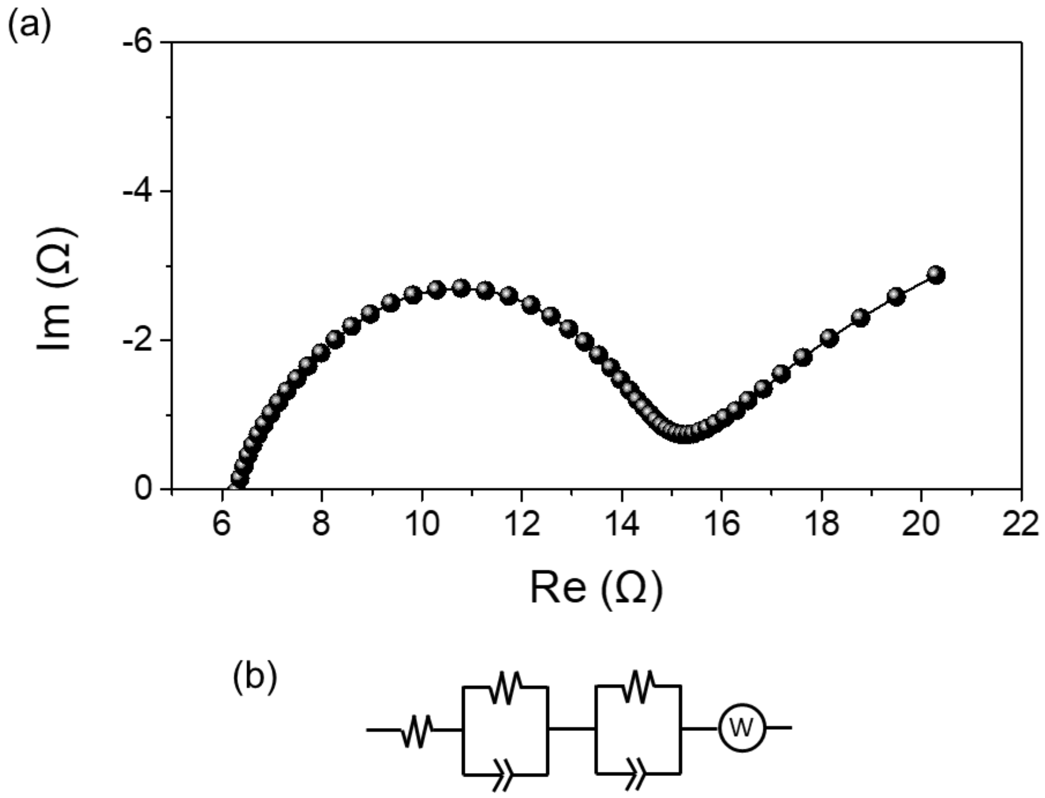

Figure S1. (a) Impedance spectra of fresh ML2032 coin-cell sample (b) Equivalent circuit example for interpreting the impedance spectra.

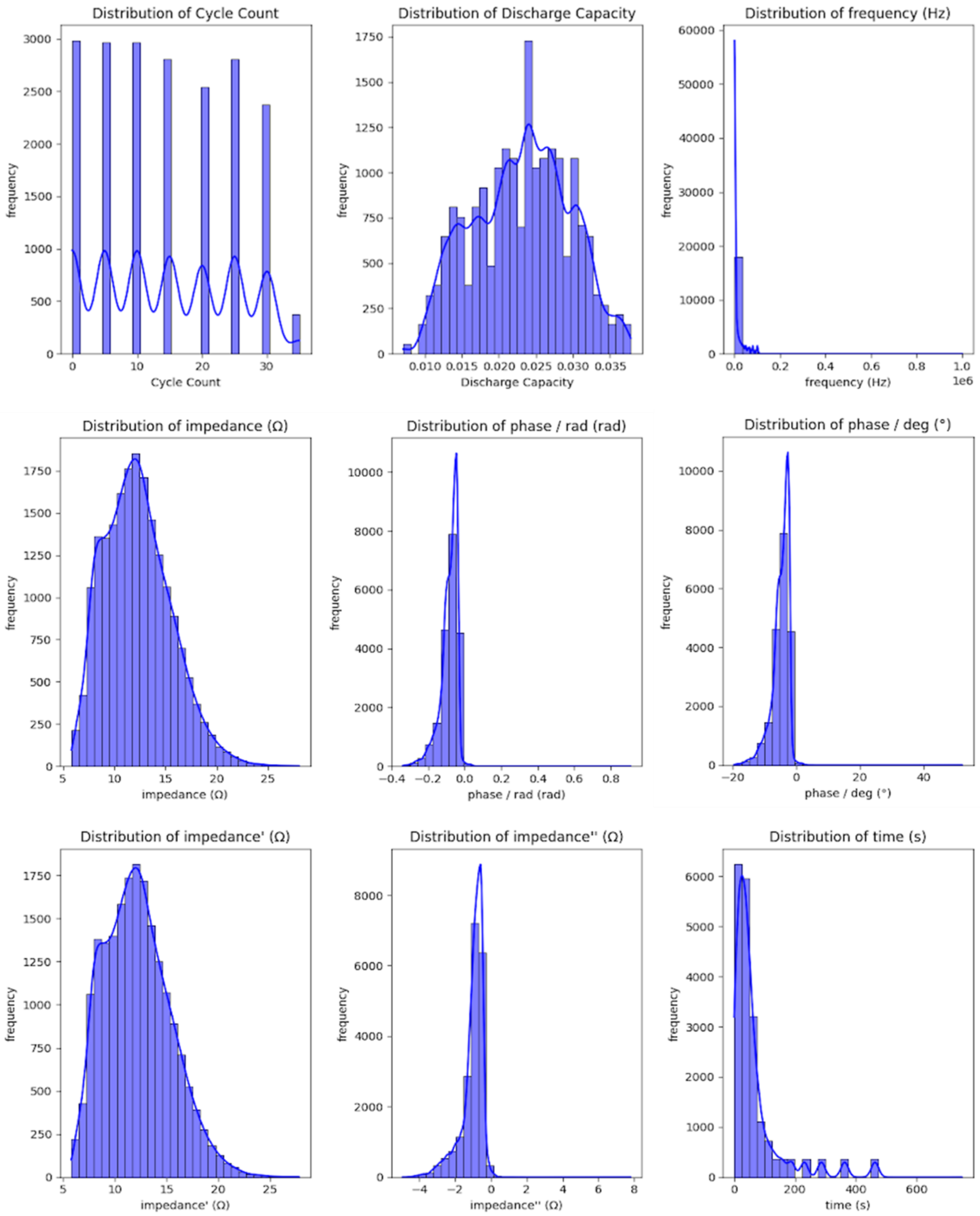

Figure S2. Data distribution of collected impedance data from ML2032. (phase/rad and phase/deg have same value with different unit)

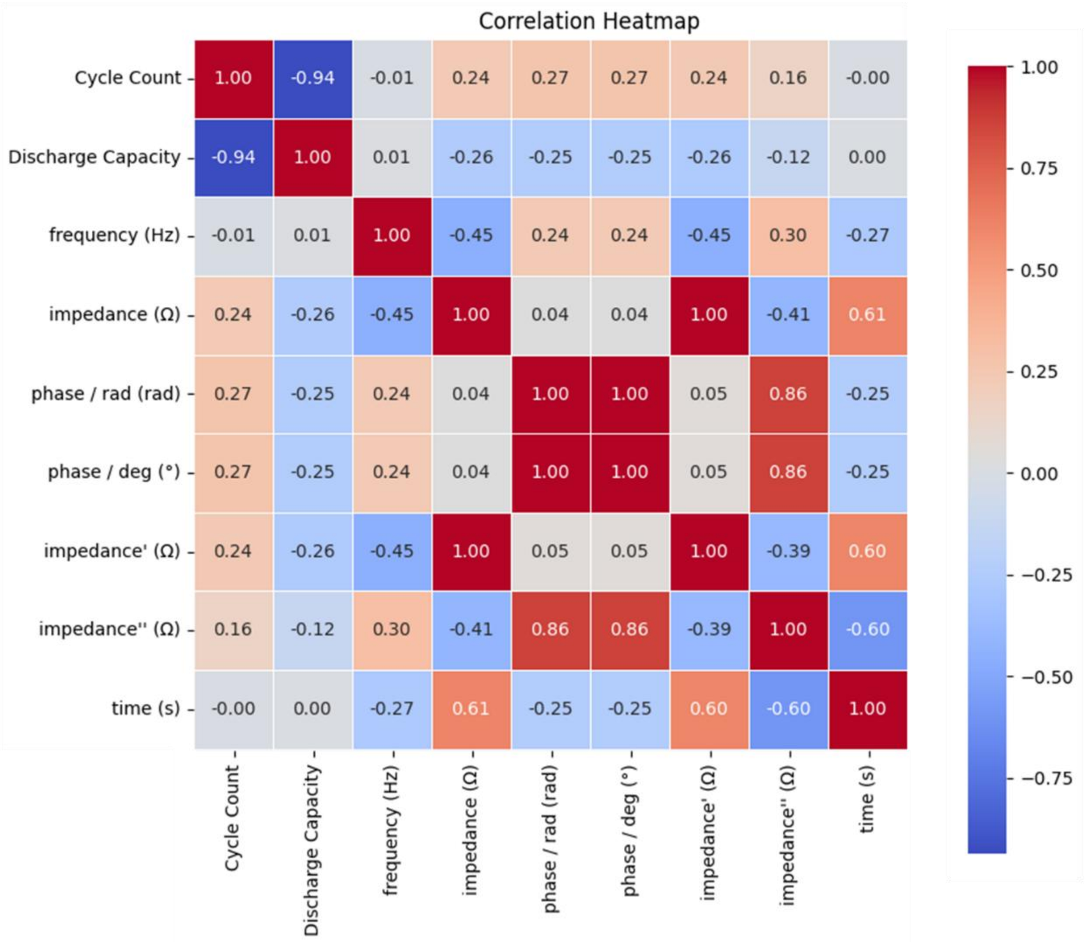

Figure S3. Result of correlation analysis in detail. (phase/rad and phase/deg have same value with different unit)

ML model	Explanation
**XGBoost**	XGBoost (Extreme Gradient Boosting) is an advanced implementation of the gradient boosting framework, where models are built sequentially, improving each subsequent model upon the errors of the previous model. To handle large datasets efficiently, XGBoost uses an approximate algorithm to find the best split points. This involves grouping data into quantile bins, reducing the need to scan through all data points, and speeding up tree-building.<br>XGBoost uses the following objective function:<br>$$Objective = \sum_{i=1}^{n} L(y_i, \hat{y}_i) + \sum_{k=1}^{K} \Omega(f_k)$$<br>In the loss function, $L(y, \hat{y})$ is a differentiable loss function and $\Omega(f_k)$ is the regularization term that penalizes the complexity of each tree f_k.
**CatBoost**	CatBoost (Categorical Boosting) is a machine learning model based on gradient boosting and is specifically optimized to handle categorical features efficiently. In traditional boosting, all data is used at each iteration, but the model focuses on learning from the residual errors of previous iterations, which can lead to overfitting in some cases. CatBoost implements "ordered boosting" method, where the model calculates the loss for each data point without using that specific data point in training to avoid overfitting to the training set.
**Random Forest**	The Random Forest model is an ensemble learning algorithm, meaning it builds a "forest" of many decision trees and combines their outputs.

	Each tree in the forest is trained on a subset of data, and their predictions are aggregated to make a final prediction. Random Forest uses bagging to create diversity among trees. It involves randomly sampling data points with replacement to create different subsets of the training data. Each tree is trained on a unique subset, making the trees diverse and reducing overfitting.
**LightGBM**	LightGBM (Light Gradient Boosting Machine) is a highly efficient and scalable gradient boosting framework designed to handle large datasets and high-dimensional data with speed and accuracy. LightGBM uses GOSS to improve efficiency by reducing the number of data instances used in each iteration. It selects instances with large gradients (indicating higher error) to focus on hard-to-predict samples and randomly samples from smaller gradients to maintain representativeness. And EFB, which is unique method of LightGBM, is a feature reduction technique that combines mutually exclusive features (those rarely non-zero together) into single features, reducing the number of features and improving computational efficiency. This is especially useful for sparse data.

Table S1. Detailed explanation for machine learning model.

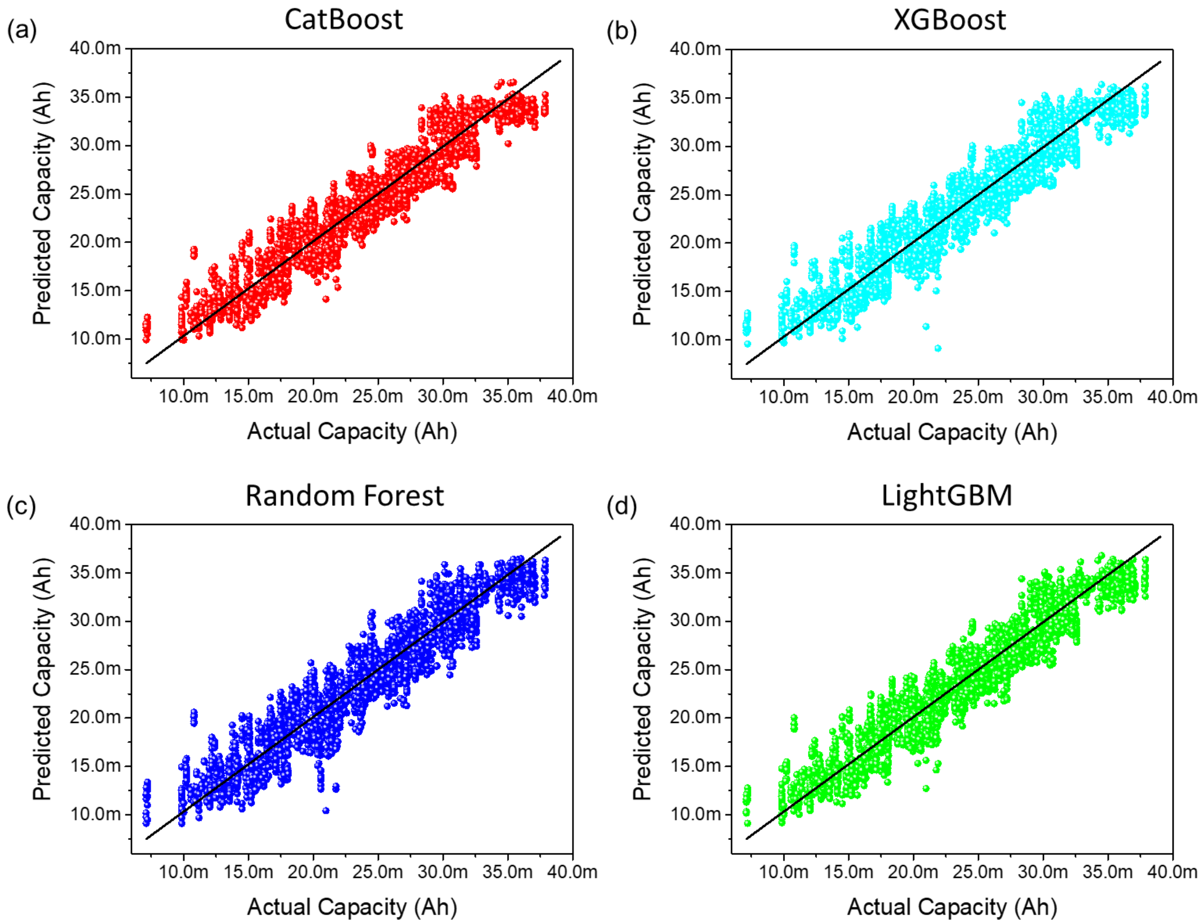

Figure S4. Graph of Actual capacity versus predicted capacity of ML2032 with (a) Catboost (b) XGBoost (c) Random Forest and (d) LightGBM, trained using single data as one row in tabular impedance data.

ML model	Mean Squared Error	R-squared
**XGBoost**	8.469×10^{-6}	0.802
**CatBoost**	8.682×10^{-6}	0.797
**Random Forest**	8.273×10^{-6}	0.806
**LightGBM**	9.234×10^{-6}	0.784

Table S2. Prediction accuracy of machine learning models trained using single data as one row in tabular impedance data.

Data Number	Frequency (Hz)	Data Number	Frequency (Hz)	Data Number	Frequency (Hz)
1	100000	19	1650.4	37	25.621
2	79612	20	1313.9	38	19.647
3	63380	21	1046	39	14.914
4	50548	22	832.75	40	11.2
5	40171	23	662.97	41	8.3137
6	31980	24	527.8	42	6.0959
7	25460	25	420.19	43	4.4116
8	20269	26	334.52	44	3.149
9	16137	27	266.32	45	2.2154
10	12847	28	212.02	46	1.5353
11	10227	29	168.79	47	1.0478
12	8142.3	30	134.38	48	0.70412
13	6482.2	31	106.98	49	0.46612
14	5160.6	32	85.17	50	0.30424
15	4108.4	33	67.805	51	0.19611
16	3270.8	34	53.812	52	0.12512
17	2603.9	35	42.377	53	0.079245
18	2073	36	33.097	54	0.05

Table S3. Frequency corresponding to each data number in impedance spectra.

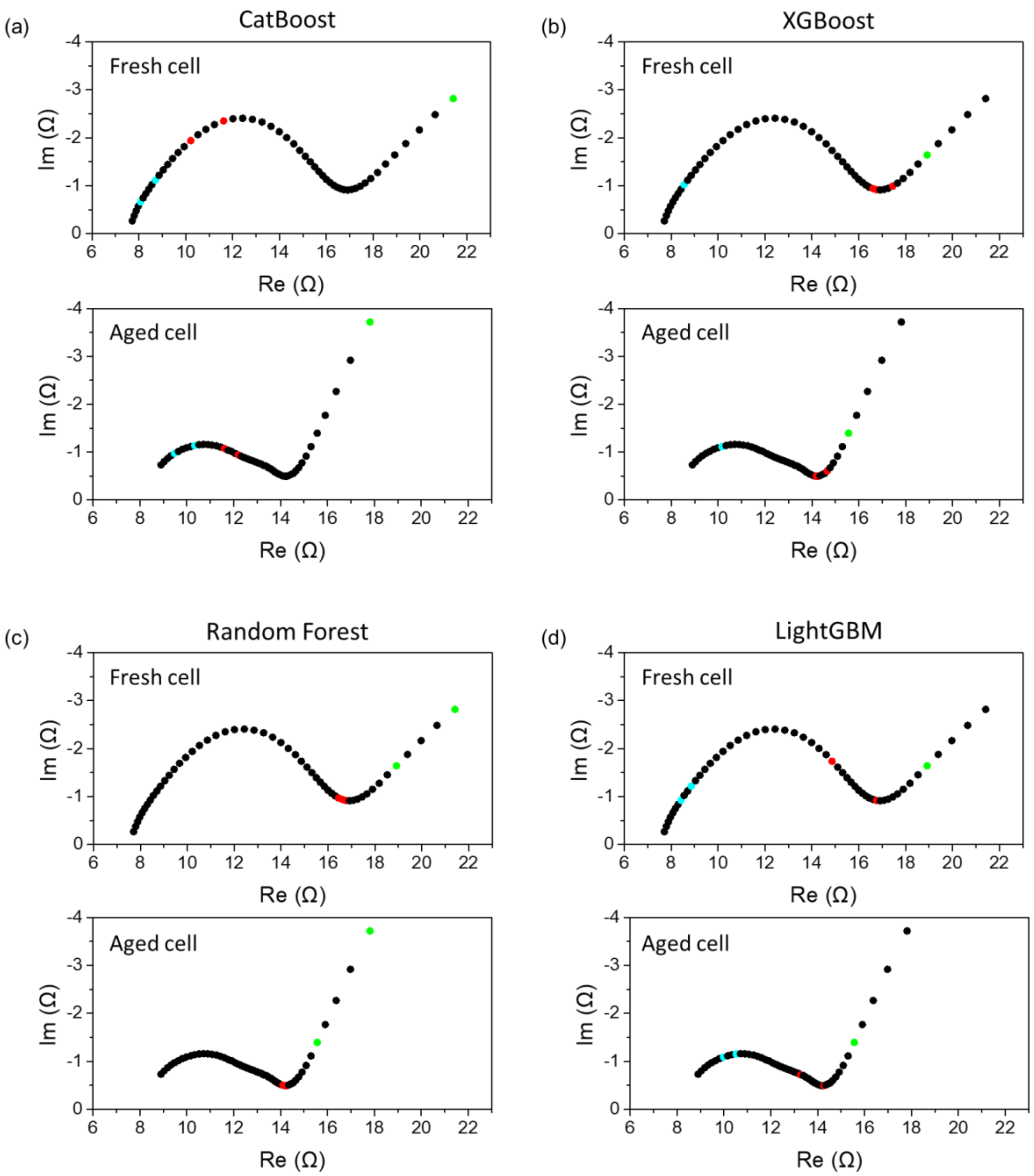

Figure S5. Top 5 contributing data points from (a) Catboost (b) XGBoost (c) Random Forest and (d) LightGBM on impedance spectra of fresh cell and aged cell. The colored data points represent the top five feature importance, with cyan indicating the high-frequency domain, red the mid-frequency domain, and green the low-frequency domain.

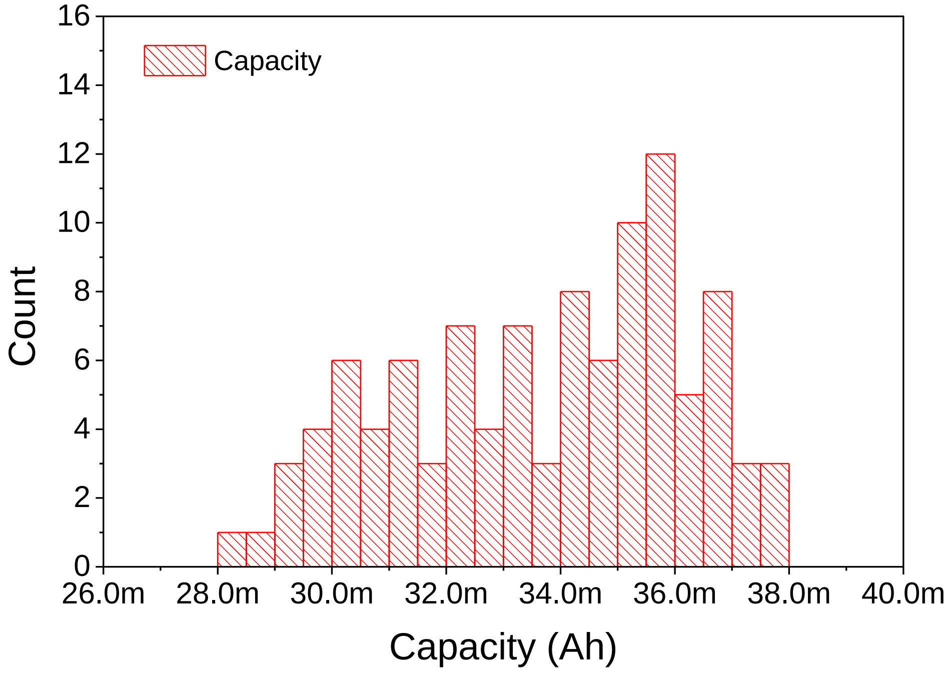

Figure S6. Initial capacity distribution of ML2032 cells
